# Efficacy of Hospital at Home in Patients with Heart Failure: A Systematic Review and Meta-Analysis

**DOI:** 10.1371/journal.pone.0129282

**Published:** 2015-06-08

**Authors:** Amro Qaddoura, Payam Yazdan-Ashoori, Conrad Kabali, Lehana Thabane, R. Brian Haynes, Stuart J. Connolly, Harriette Gillian Christine Van Spall

**Affiliations:** 1 Department of Medicine, Queen’s University, Kingston, Ontario, Canada; 2 Department of Medicine, McMaster University, Hamilton, Ontario, Canada; 3 Dalla Lana School of Public Health, Division of Epidemiology, University of Toronto, Toronto, Ontario, Canada; 4 Department of Clinical Epidemiology and Biostatistics, McMaster University, Hamilton, Ontario, Canada; 5 Population Health Research Institute, McMaster University, Hamilton, Ontario, Canada; University of Naples Federico II, ITALY

## Abstract

**Background:**

Heart failure (HF) is the commonest cause of hospitalization in older adults. Compared to routine hospitalization (RH), hospital at home (HaH)—substitutive hospital-level care in the patient’s home—improves outcomes and reduces costs in patients with general medical conditions. The efficacy of HaH in HF is unknown.

**Methods and Results:**

We searched MEDLINE, Embase, CINAHL, and CENTRAL, for publications from January 1990 to October 2014. We included prospective studies comparing substitutive models of hospitalization to RH in HF. At least 2 reviewers independently selected studies, abstracted data, and assessed quality. We meta-analyzed results from 3 RCTs (n = 203) and narratively synthesized results from 3 observational studies (n = 329). Study quality was modest. In RCTs, HaH increased time to first readmission (mean difference (MD) 14.13 days [95% CI 10.36 to 17.91]), and improved health-related quality of life (HrQOL) at both, 6 months (standardized MD (SMD) -0.31 [-0.45 to -0.18]) and 12 months (SMD -0.17 [-0.31 to -0.02]). In RCTs, HaH demonstrated a trend to decreased readmissions (risk ratio (RR) 0.68 [0.42 to 1.09]), and had no effect on all-cause mortality (RR 0.94 [0.67 to 1.32]). HaH decreased costs of index hospitalization in all RCTs. HaH reduced readmissions and emergency department visits per patient in all 3 observational studies.

**Conclusions:**

In the context of a limited number of modest-quality studies, HaH appears to increase time to readmission, reduce index costs, and improve HrQOL among patients requiring hospital-level care for HF. Larger RCTs are necessary to assess the effect of HaH on readmissions, mortality, and long-term costs.

## Introduction

Heart failure (HF) is associated with substantial morbidity and mortality, and is the commonest cause of hospitalization in patients over the age of 65 in developed countries [1]. The incidence of HF hospitalizations has tripled in less than 3 decades [2]. Patients hospitalized for HF face a higher risk of death and a reduced health-related quality of life (HrQOL) than those not hospitalized [3]. Patients with chronic HF typically have a relapsing and remitting course with an overall decline in health status requiring frequent hospitalizations during the advanced stage of their disease. HF hospitalizations burden the health care system, accounting for more than 70% of the annual cost of HF care [4,5]. Furthermore, care processes as patients transition from hospital to home are often suboptimal, and account for a significant proportion of readmissions and health care utilization [6]. With the growing prevalence of HF, there is substantial motivation to explore models of care other than routine hospitalization (RH) that may improve HrQOL, facilitate seamless transitions from the hospital to the post-discharge phase, and improve clinical outcomes.

Hospital at home (HaH) is the delivery of hospital ward-level care in the patient’s home as a substitute for routine hospitalization. Patients who have clinical indications for admission to a hospital ward are offered monitoring, face-to-face clinical care from nurses and physicians, diagnostic testing (e.g. laboratory investigations, electrocardiograms, radiography), and intravenous (IV) medication in their homes. HaH has been proposed as an alternative to RH in patients with acute illness [7]. Potential benefits include fewer in-hospital complications and adverse events, increased patient and caregiver satisfaction, improved functional outcomes, conservation of hospital resources, and cost effectiveness [7–12]. Effects of HaH in the literature are conflicting due to variances in patient populations, interventions, and definitions. A systematic review of 26 randomized controlled trials (RCTs) in patients with general medical and surgical conditions found that a brief hospital stay followed by discharge to the patient’s home for ongoing hospitalization improved patient satisfaction, but had no clear effect on readmissions or costs [13]. Another systematic review of 10 RCTs of HaH as an alternative to RH in patients with general medical and surgical conditions found that HaH improved patient satisfaction, decreased mortality, and reduced costs [14]. These reviews included patients with stroke and pulmonary conditions such as chronic obstructive pulmonary disease (COPD), and typically did not include patients with HF.

The effectiveness of HaH and other substitutive models of care in patients requiring hospital-level care for decompensated HF is unclear, and has not previously been reviewed. We aimed to synthesize the literature to address the effect of HaH on clinical, patient-centered, and health care system cost outcomes in HF.

## Methods

We followed the Preferred Reporting Items for Systematic Reviews and Meta-Analyses (PRISMA) and Meta-Analysis Of Observational Studies in Epidemiology (MOOSE) statements for reporting our systematic review and meta-analysis [15,16].

### Data sources and searches

We conducted a systematic search of MEDLINE, Embase, CINAHL, and CENTRAL from 1 January 1990 to 28 May 2014, guided by a librarian, and using a combination of keywords and medical subject headings with no language restrictions. The search strategy for each database is available in S3, S4, S5, and S6 Tables. In addition, we searched abstracts, conference proceedings, and reference lists for other potentially relevant articles. We also screened weekly e-mail auto-alerts for any new citations in MEDLINE, Embase, and CENTRAL from 28 May 2014 to 21 October 2014.

### Study selection

We specified inclusion and exclusion criteria a priori. We included prospective studies that tested substitutive care models in patients who required hospitalization for decompensated HF. We assessed models of care that aimed to avoid or reduce RH with one of the following predefined interventions: 1) admission avoidance schemes that provided active treatment in the patient’s home as a means of avoiding hospital inpatient care [14]; 2) early discharge schemes that facilitated early discharge from the hospital with active hospital-level treatment in the patient’s home [13]; and 3) day hospital care schemes that avoided admission to the hospital by providing hospital-level care in an outpatient setting during daytime hours [17]. We considered three broad categories of outcomes: clinical (e.g. hospital readmissions, mortality, length of stay in care, emergency department [ED] visits), patient-centered (e.g. functional status, HrQOL, caregiver or family burden, patient satisfaction), and cost (e.g. health system costs) outcomes. We used translators to screen and translate potentially eligible non-English abstracts and articles.

We excluded studies that did not present results for patients with a principal diagnosis of HF, those in which the intervention did not offer hospital-level care as a substitute to routine hospitalization, and those that only offered services following discharge from hospital (e.g. tele-medicine, transitional care services). We excluded retrospective studies.

Two authors (AQ, PYA) independently screened titles and abstracts for eligibility. Three authors (AQ, PYA, HV) reviewed potentially eligible abstracts and full-text articles for inclusion, and resolved disagreements by consensus.

### Data extraction and quality assessment

Three authors (AQ, PYA, HV) independently extracted details pertaining to methods, participants, interventions, and outcomes. They resolved conflicts by consensus.

Two authors (AQ and PYA) independently assessed the risk of bias for included studies using the criteria in the *Cochrane Handbook for Systematic Reviews of Interventions* [18]. We scored the risk of bias for each of the criteria as ‘high’, ‘low’, or ‘unclear’ for RCTs, and as ‘adequate’ or ‘inadequate’ for non-randomized studies (S7 Table). The “other bias” category included pre-specified assessment criteria, such as: baseline imbalance, confounders, contamination, co-intervention, lack of standardized protocol, and potential conflicts of interest. A third author (HV) resolved disagreements. If published studies had insufficient information for data extraction or to assess the risk of bias, we made attempts to contact authors via electronic and telephone correspondence.

Our primary outcomes were (1) mortality, and (2) hospital readmissions. Secondary outcomes were other clinical, patient-centered, and cost outcomes.

### Data synthesis and analysis

We assessed inter-rater reliability for article screening and outcome extraction using the kappa index. We pooled outcomes if they were similar and derived from a similar study design, using risk ratios and mean differences as summary measures. We analyzed observational studies separately from RCTs. We evaluated heterogeneity of effect size across studies by using the I^2^ index [19]. We set the cut-off points for low, moderate, and high I^2^ values at 25%, 50%, and 75%, respectively. We did not calculate pooled estimates for studies with I^2^ values ≥75%. We applied the DerSimonian-Laird random effects model with a Knapp-Hartrung modification for pooled estimates as recent methodological research has shown that the standard DerSimonian-Laird random effects approach can produce narrow confidence intervals when the number of studies is small or when substantive heterogeneity is present [20,21]. To facilitate the pooling, we converted mean difference (MD) for HrQOL measurements to standard deviation (SD) scale. We considered meta-regression to identify sources of heterogeneity, but the small number of studies precluded this.

We performed a narrative review for outcomes that could not be pooled due to differences in outcome measure or when results in the individual study were skewed. We did not use a funnel plot to illustrate publication bias given the small number of included studies (n<10). Since some studies did not report SDs, we calculated them using confidence intervals (CI) or P values. Statistical significance was taken at the two-tailed 5% level. We used the R package metaphor for analyses [22].

## Results

### Identification and characteristics of studies

**Fig 1:**
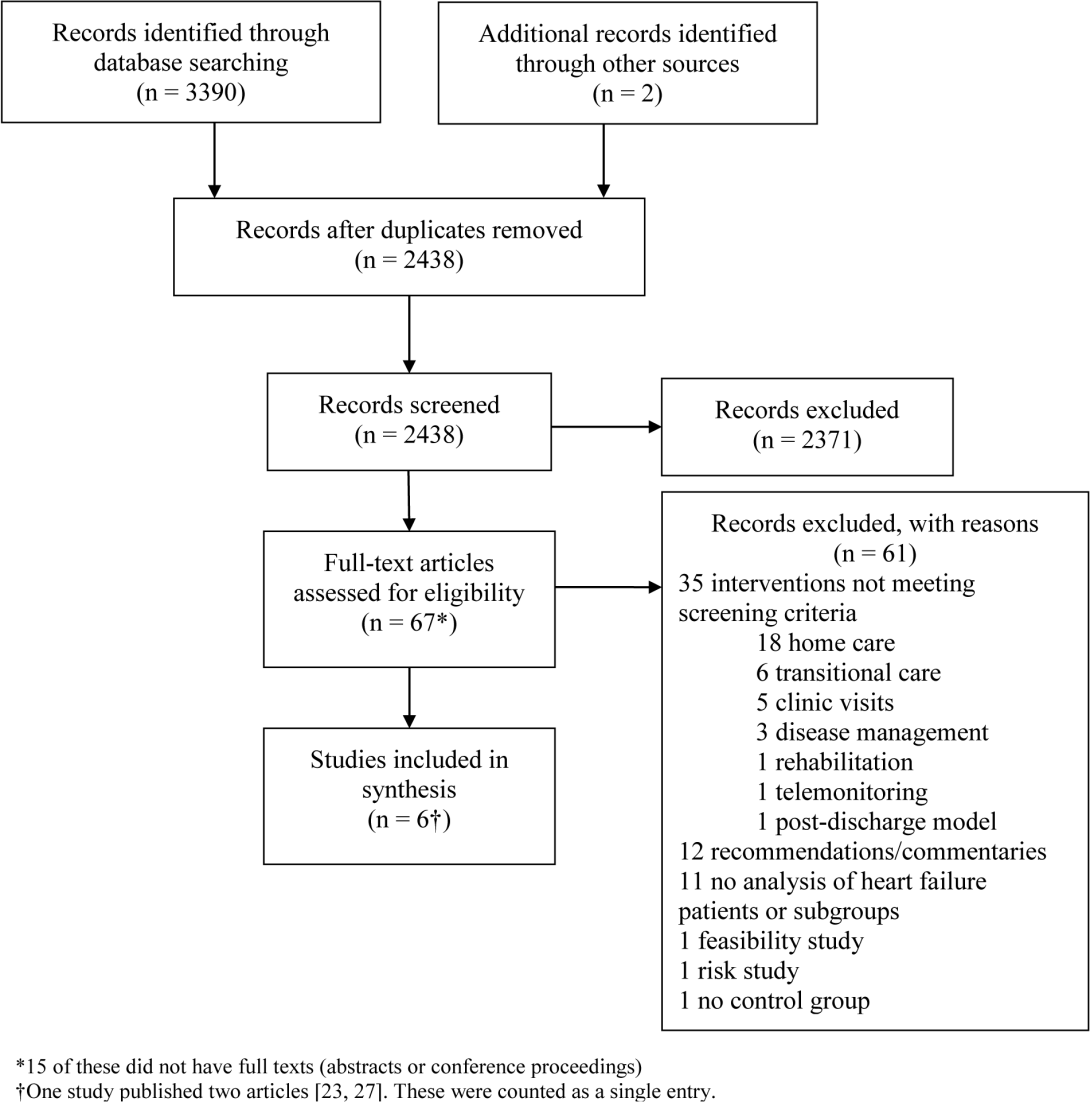
Summary of evidence search and selection.

The systematic literature search resulted in 2436 unique records (Fig 1). After removing duplicates and screening abstracts, we assessed 67 full-text articles for potential eligibility. Of these, we excluded 35 whose interventions did not meet our inclusion criteria. We further excluded 29 articles that were not prospective studies or did not include HF patients. Only 6 studies, including 3 randomized controlled trials (RCTs) (n = 203) and 3 observational studies (n = 329) were eligible for inclusion [23–28]. We did not identify additional eligible studies in either the re-run search or the weekly e-mail auto-alerts ending on 21 October, 2014.

The 6 studies originated from Spain (4 trials), Italy (1 trial), and Sweden (1 trial). All studies recruited patients from the ED, hospital ward, or community. All studies considered inpatient hospital care as the comparison group. Three RCTs and 2 observational studies tested the effectiveness of ‘hospital avoidance’ HaH schemes. 1 observational study tested a day hospital scheme. We did not find any studies that assessed ‘early discharge’ HaH schemes. One study [25] had a subsequent publication with long-term follow-up results [29]; when there was an overlap in outcomes reported in the 2 articles, we used the results in the follow-up article for our analysis. See Table 1 for study characteristics and S7 Table for details on the methods, patients, interventions, and outcomes reported in each included study.

**Table 1:** Characteristics of included studies.

Study, year (reference)	Design, Country	Group, n	Intervention*	Intensity	Mean age	Male (%)	NYHA class	EF	Comorbidities	Risk of bias*
Bechich et al, 2000 [23]	PC, Spain	HaH, 110†	Five levels of care based on patient needs; telephone access to HaH staff	Low	85	NR	NR‡	NR	COPD 30, RF 14, HTN 10, DM 7, RI 9, stroke 4	3/7
de Zuazu et al, 2003 [24]	PC, Spain	HaH, 158†	Daily nursing visits and doctor visits at least bidiurnally; telephone access to HaH staff	Medium	78	91 (58)	II-II: 26, II: 103, IV: 29	NR	NR	3/7
Mendoza et al, 2009 [25]	RCT, Spain	HaH, 37 RH, 34	Daily specialist nurse visits and doctor visits at least bidiurnally. Lab values and ECGs done at home; radiographs and echocardiograms at hospital	Medium	79	52 (73)	II: 42, III: 29	<45%: 24	AF 37, cancer 7, COPD 23, DM 23, HCE 29, HTN 61, RF 20	3/7
Patel et al, 2008 [26]	RCT, Sweden	HaH, 13 RH, 18	Daily or bidiurnal specialist nurse visits with tests, care, and self-management education provided at home. Consultations with cardiologists and other specialists were available	Medium	78	21 (68)	II: 1, III: 29, IV: 1	34%	AF 18, DM 12, HTN 16, IHD 21, RD 11, stroke 7, RF 1, VD 7	4/7
Roig et al, 2006 [27]	PC, Spain	Day hospital, 61†	Tests, care, and self-management education provided at day hospital. Specialist nurse and cardiologist decided which patients required IV therapy: monthly, biweekly, or IV portacath. Monitoring for decompensation and changes to drug regimen were added in the second year with nurse home visits	Medium	64	56 (92)	III: 23, IV: 38	23%	IHD 34, DC 24, VD 6, RC 1	3/7
Tibaldi et al, 2009 [28]	RCT, Italy	HaH, 48 RH, 53	Doctor and nurse home visits to run all tests, provide care, and teach self-management. OT, PT, counseling, and surgical pressure sore treatment were consulted as necessary. Doctors and nurses met daily to discuss patient needs	High	81	52 (51)	III: 66, IV: 35	<40%: 40	ACS 6, AF 31, DC 11, HC 37, IHD 27, VD 16	6/7

*Details in S7 Table; for risk of bias, numerator indicates the number of “low risk” categories as per the Cochrane tool (higher fractions have a lower risk of bias).

†Patients acted as their own controls.

‡NYHA class only reported for patients who died: 5 class IV, 4 class III, and one class II.

ACS = acute coronary syndrome; AF = atrial fibrillation; COPD = chronic obstructive pulmonary disease; DC = dilated cardiomyopathy; DM: diabetes mellitus; ECG = electrocardiogram; EF = ejection fraction; HaH = hospital at home; HC = hypertensive cardiopathy; HCE = hypercholesterolemia; HTN = hypertension; IHD = ischemic heart disease; IV = intravenous; NR = not reported; NYHA = New York Heart Association; OT = occupational therapy; PC = prospective cohort; PT = physical therapy; RC = restrictive cardiomyopathy; RCT = randomized controlled trial; RD = respiratory disease; RF = renal failure; RH = routine hospitalization; RI = respiratory infection; SD = standard deviation; VD = valve disease.

### Quality of included studies

The risk of bias of included studies is summarized in Table 1, with details in S7 Table. Three of 6 studies were published RCTs [25,26,28], but allocation concealment was not adequately described in 2 [25,26]. No studies blinded participants and outcome assessors. We determined 2 studies to have incomplete outcome data in their control groups as one had a 20.6% dropout rate [25] and the other had 17% who withdrew consent [26], creating an imbalance between the intervention and control groups. Three studies involved possible lack of protocol standardization [24,25,27]. Overall, the RCTs were modest in quality, at best. The observations studies were limited in quality by virtue of their study design, which did not account for confounders and which precluded accurate assessment of effect size.

### Inter-rater reliability

There was excellent agreement in the screening process (kappa, 0.82 [95% CI, 0.63 to 1.00]), and outcome extraction (kappa, 0.98 [95% CI, 0.95 to 1.00]).

### All-cause mortality

**Fig 2:**
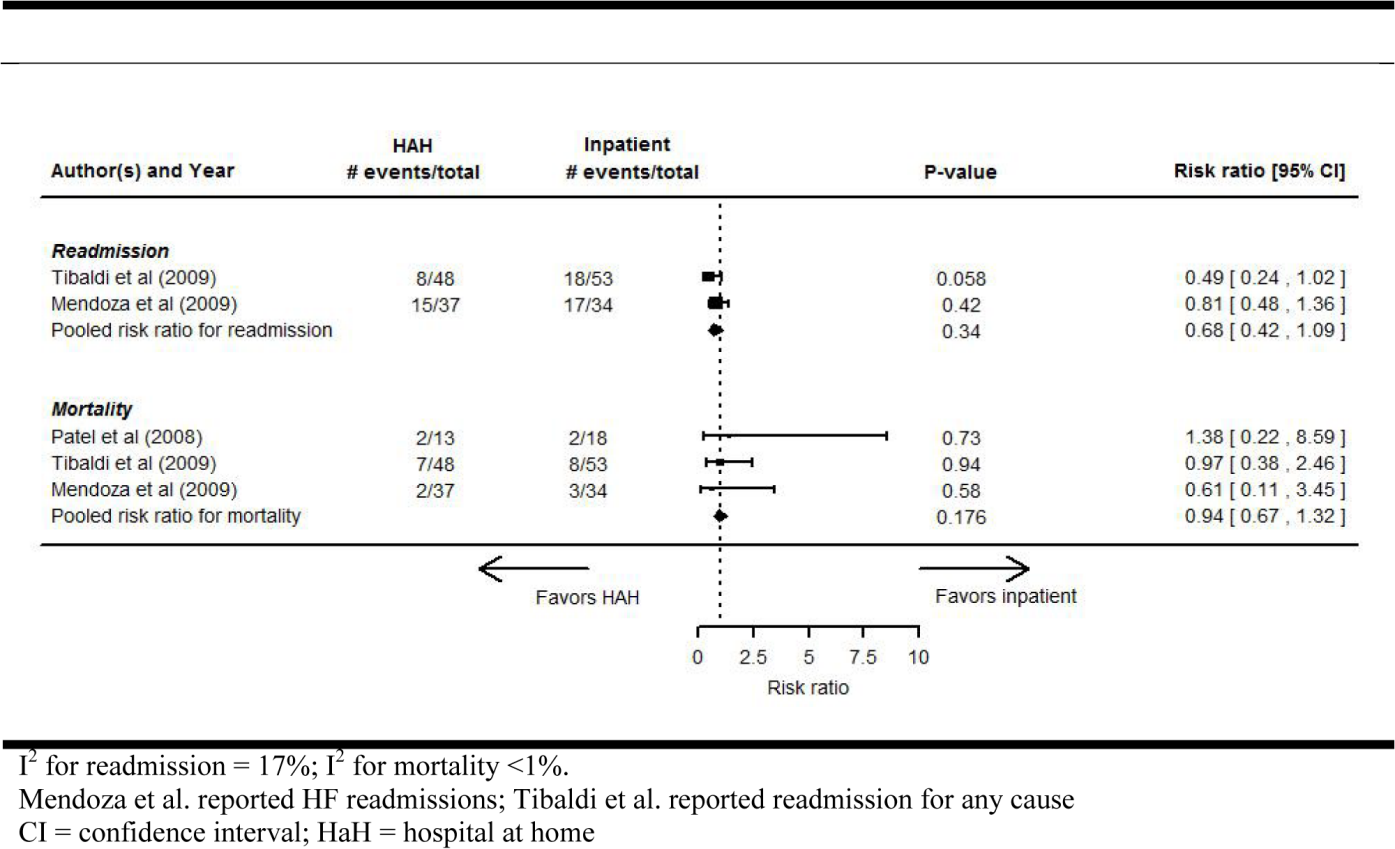
Hospital readmissions and all-cause mortality for HaH compared with inpatients in randomized controlled trials.

Among the 3 RCTs (203 patients) that reported all-cause mortality (Fig 2), there was no significant difference between the HaH and RH groups (RR, 0.94 [95% CI, 0.67 to 1.32]; P = 0.176; I^2^ <1%). One observational study [24] reported all-cause mortality, with results that favored the HaH group. In this study, 6 (3.8%) of the 158 HaH patients died, while 132 (9.7%) of the 1358 concurrent control patients died (P<0.05).

### Readmissions

Two RCTs reported the number of patients readmitted to hospital, either for HF [25] or any cause [28] (Fig 2). Although the pooled estimate was in favor of HaH, results were not statistically significant (RR, 0.68 [95% CI, 0.42 to 1.09]; P = 0.34; I^2^ = 17%). One RCT reported the average number of readmissions per patient, but this was not pooled with the other RCTs due to differences in outcome measure [26]; this study demonstrated a non-significant reduction of the average readmissions per patient in the HaH group compared to the control (mean [SD] of 0.5 [0.8] versus 0.6 [0.8], respectively).

**Fig 3:**
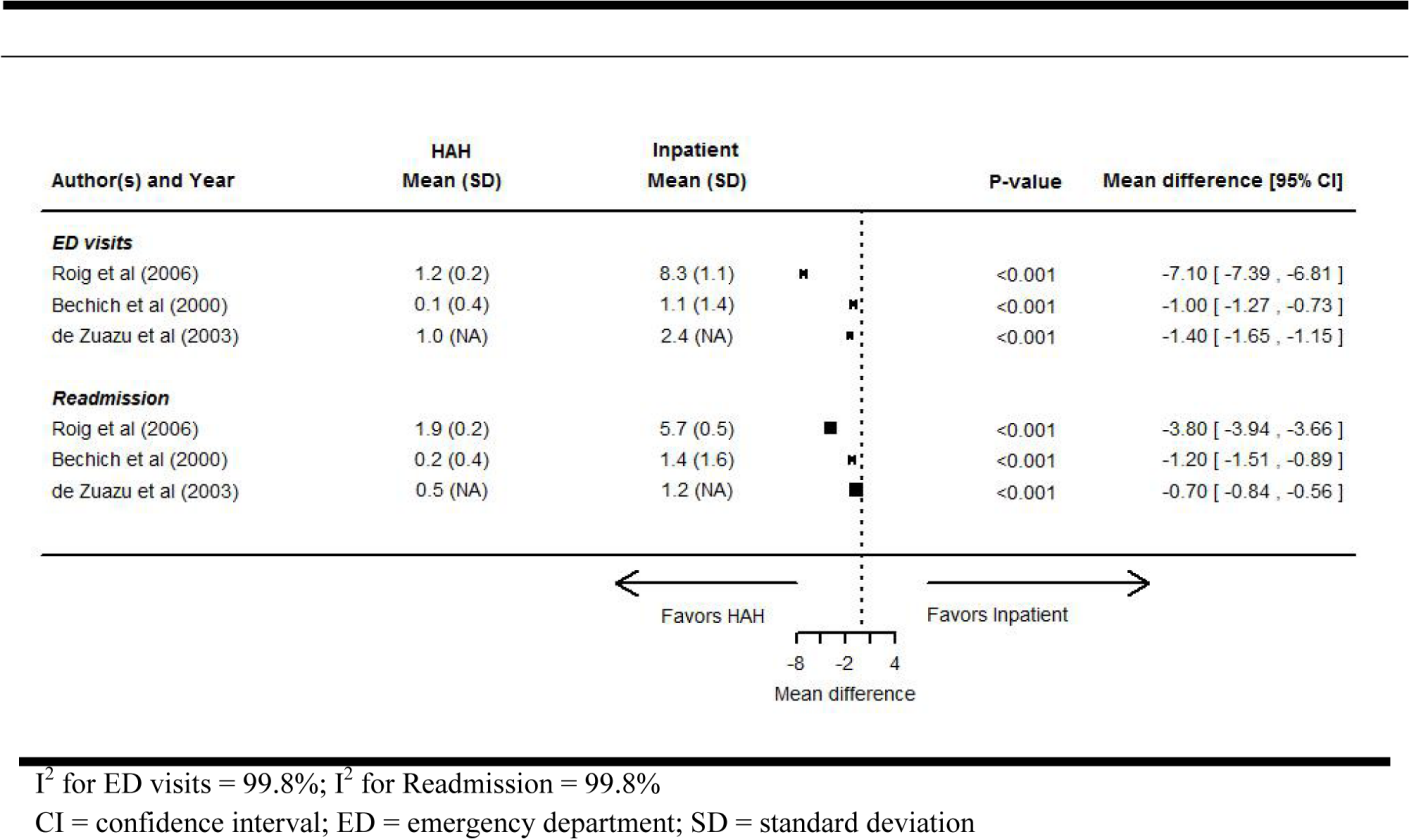
Hospital readmissions and emergency department visits per patient in substitutive versus inpatients in observational studies.

Three observational studies, including one that tested the efficacy of a day hospital scheme, reported average readmissions per patient [23,24,27] (Fig 3). All 3 studies reported a significant reduction in favor of substitutive hospital care (MD range, -3.80 to -0.70 readmissions per patient). We did not pool these results due to substantial heterogeneity between the studies (I^2^ = 99%).

### Average number of ED visits per patient

All 3 observational studies reported average ED visits per patient [23,24,27] (Fig 3). Individual studies demonstrated a significant reduction in favor of substitutive hospital care (MD range, -7.10 to -1.00 visits per patient). We did not pool these results due to substantial heterogeneity across studies (I^2^ = 99%).

One RCT [26] reported the average number of ED visits per patient, but there was no significant difference between the HaH and RC group (mean [SD] of 0.3 [0.5] versus 0.3 [0.6], respectively).

### Time to first readmission

Two RCTs reported the number of days between discharge and the first readmission (S1 Fig) [26,28]. HaH significantly increased time to readmission (MD, 14.13 days [95% CI 10.36 to 17.91]; P = 0.015; I^2^ <1%).

### Length of stay in index care

Two RCTs reported the average length of index stay per patient (S2 Fig) [25,28]. HaH increased the length of index stay in each study (MD, 3.00 and 9.10 days), but the degree of heterogeneity was too high to allow pooling (I^2^ = 88%).

Two observational studies reported the length of stay in index care [24,27]. One showed a significantly longer length of stay in the HaH group as compared with the concurrent control group (mean [SD] of 12.8 [6.53] days versus 9.4 [7.4] days, respectively; P<0.05), while the other reported a significant reduction in the length of index stay in the day hospital group (MD, 34 days [95% CI, 45 to 22]; P < 0.001).

### Health-related quality of life

**Fig 4:**
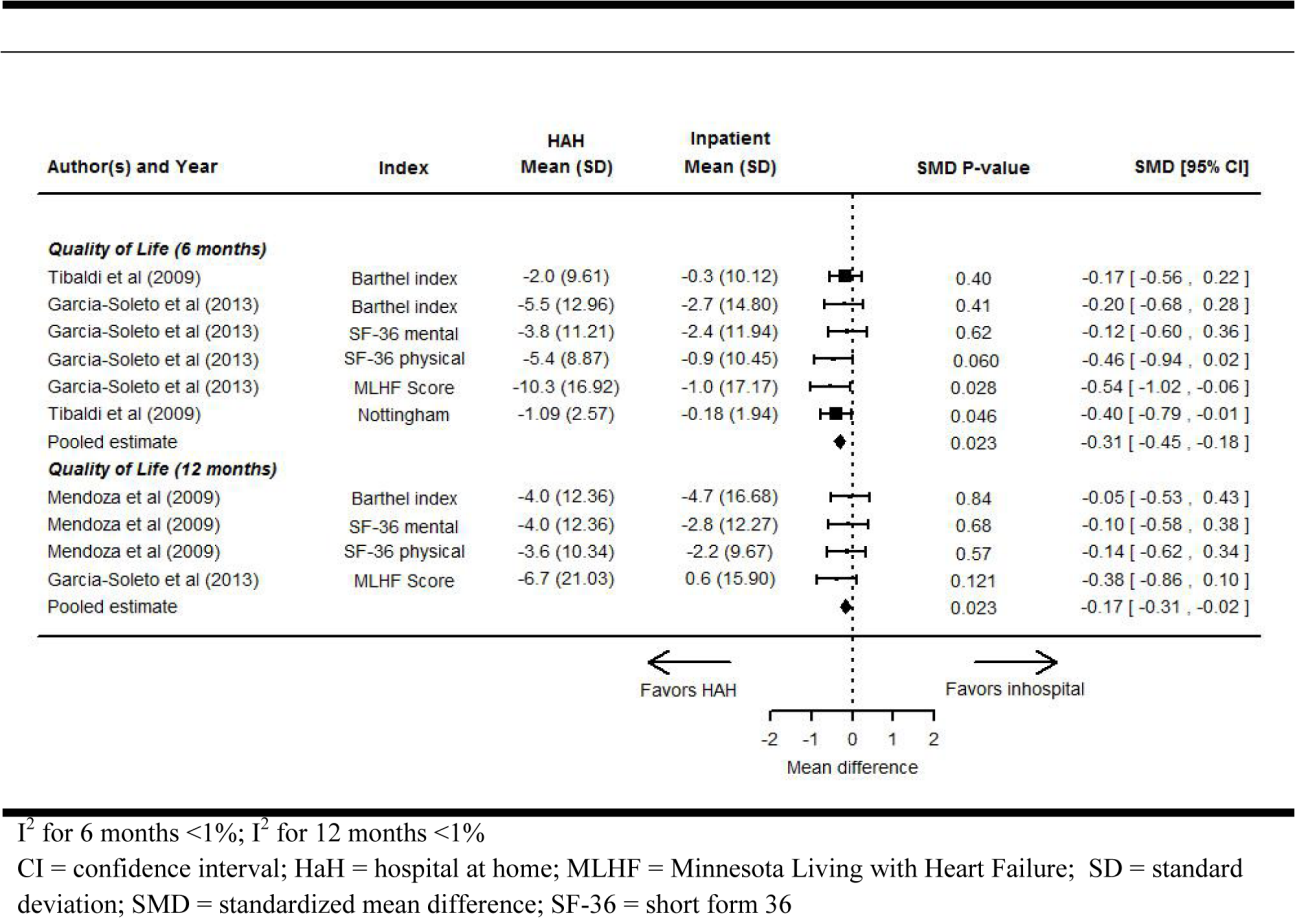
Health-related quality of life for HaH compared with inpatients at 6- and 12-month follow-up in randomized controlled trials.

Two RCTs reported HrQOL using various scales at 6-month follow-up (Fig 4) [25,28]. We converted the MD measured on these scales to standardized mean differences (SMD) and pooled the results across studies, with negative SMDs indicating improvement. HaH significantly improved HrQOL compared to RH at 6-month follow-up (SMD, -0.31 [95% CI, -0.45 to -0.18]; P = 0.023; I^2^ <1%). One study (66 patients) reported HrQOL outcomes at 12-month follow-up in 2 publications [25,29]. Meta-analysis showed a significant difference in favor of HaH (SMD, -0.17 [95% CI, -0.31 to -0.02]; P = 0.023; I^2^ <1%).

One observational study measured functional status using the Barthel Index [23], which significantly improved with HaH from a mean score of 74 at admission to 77 at discharge (P < 0.05). No SDs were reported.

### Number of readmissions

One RCT and one observational study measured the total number of readmissions [25,27]. We did not pool the results due to differences in design and outcome measurements. The results are reported in S1 Table. The RCT demonstrated similar readmission proportions between the 2 groups [25]. The observational study assessing the effectiveness of a day hospital intervention reported a reduction in the number of all-cause hospitalizations by 200 (65% decrease) in the day hospital cohort [27].

### Index care costs and follow-up costs

Three RCTs demonstrated reduced total costs in the HaH group [25,26,28]. We did not meta-analyze these results due to differences in outcome measures as well as skewed data. The results are summarized in Table 2. In all 3 RCTs, HaH significantly reduced the cost of index care per patient (P < 0.001). Two trials also reported follow-up costs at 12 months [25,26]; follow-up costs were lower in the HaH group in both of these trials, although the difference was not statistically significant in one of the trials [25]. We calculated total costs (for index and follow-up care) when not presented in the primary studies, and found that this outcome was lower in the HaH group in both studies (achieving statistical significance in the single study that reported P values) (Table 2). In addition, there was a cost benefit derived from lower long-term institutionalization in the HaH group in a single trial that reported this outcome; while no patients required institutionalization in the HaH group, 8 patients required institutionalization (mean length of stay 26 days) in the RH group, translating to a cost difference of 32,656 Euros [28]. The day hospital care model also demonstrated a cost reduction compared to RH [27].

**Table 2:** Index and follow-up care costs for RCTs comparing HaH to RH.

Reference (Study design)	Sample Size	Outcome	Substitutive Care	RH	P value
Mendoza 2009 (RCT)	37 in HaH; 34 in RH	Mean (SD) cost of index care per patient (Euros)	2541 (1334)	4502 (2153)	<0.001
	37 in HaH; 34 in RH	Mean (SD) cost during 12-month follow-up* (Euros)	3425 (4948)	4619 (7679)	0.83
	37 in HaH; 34 in RH	Mean total cost per patient at 12-months (Euros)†	5996	9121	NR
Patel 2008 (RCT)	13 in HaH; 18 in RH	Median (IQR) cost of index care per patient (Euros)	586 (334–1125)	3277 (2125–5750)	<0.001
	13 in HaH; 18 in RH	Median total cost per patient at 12-months (Euros)	2680	5750	0.050
Tibaldi 2009 (RCT)	48 in HaH; 51 in RH	Mean cost of index care per patient (Euros)	1820.92	2116.89	<0.001

*Results suggest that lower bound of cost can be negative, and should be interpreted with caution.

†We calculated this as the sum of index costs and follow-up costs for comparison purposes. Index costs included cost of stay in RH or HaH, medications, diagnostic tests, consumables including health care provider time, and transportation. Follow-up costs included clinic visits and readmissions.

HaH = hospital at home; NR = not reported; RCT = randomized controlled trial; RH = routine hospitalization; SD = standard deviation.

### Other outcomes

Several other clinical, patient-centered, and cost outcomes were reported, but could not be pooled. These outcomes were either reported by a single study or a standardized measure was not used for outcome assessment across studies. These results are described narratively and summarized in S2 Table.

On balance, clinical outcomes were either unchanged or improved with substitutive care. One RCT did not find a significant difference between the HaH and RH groups for the composite outcome of mortality and readmission due to HF or another cardiovascular event [25]. The observational study examining a day hospital model demonstrated a significant reduction in the number of patients with more than 3 admissions per year [27].

HaH improved patient-centered outcomes. One RCT demonstrated significant improvements in depression and nutritional status at 6 months in the HaH group, with non-significant improvements in cognitive status [28]. Another study (not reported in S2 Table) employed a patient survey and found that 106 (96%) patients were either very satisfied or satisfied with HaH care [23]. No study mentioned adjusting for death as a competing outcome.

## Discussion

This systematic review of 6 prospective studies demonstrates that HaH–provision of hospital-level care by health care professionals in the patient’s home–may confer several benefits over RH to patients with HF. However, definitive conclusions are limited by the quantity and quality of published studies. Pooled data from RCTs revealed that HaH increased time to first readmission, but this effect was dominated by a single study. Although HaH demonstrated a trend to decreased readmissions in the 3 RCTs, the effect was not statistically significant, and some may argue that this is a more relevant outcome than time to first readmission. HaH did not substantially influence all-cause mortality. However, with only 203 patients in the combined RCTs, there would have been inadequate power to demonstrate a statistically significant difference in readmissions and mortality. HaH reduced readmissions and ED visits per patient in the observational studies, but the internal validity of these studies is limited. Thus, the effect of HaH on mortality and readmissions in HF remains unclear, and merits investigating in a larger well-designed trial.

Analysis of RCTs revealed that HaH improved HrQOL at 6 months and 12 months. The decline in functional capacity and QOL after hospitalization for HF is well recognized, and is associated with undesirable outcomes such as nursing home placement, rehospitalization, and death [3,30]. The mechanisms of functional decline following RH may be related to restricted mobility, loss of muscle mass with ensuing frailty, situational depression or delirium, and adverse events [31,32]. The improvement in HrQOL among HaH patients may be facilitated by treatment in a familiar environment, greater independence, and less technically oriented care [11].

Compared to RH, the cost of index care was significantly lower in the HaH group in all 3 RCTs. The cost of 12 month follow-up care was lower in the HaH group in both RCTs that reported this outcome, but it is not clear that the follow-up costs accounted for death as a competing risk. However, since the risk of death in the HaH group was (non-significantly) lower than that in the RH group, cost-reduction in the HaH group is unlikely to be related to deaths. The reduction in index costs is an important benefit of HaH, in light of the economic burden of HF hospitalization on health care systems. Substitutive models of care that can reduce costs while improving clinical outcomes merit implementation, but the long-term costs of HaH for HF remain unclear. Furthermore, none of the primary studies reported indirect costs (e.g. overhead) and cost-utility analyses were not presented.

Our findings highlight the potential benefits of a HaH model in managing patients with decompensated HF. In this model, patients receive hospital ward-level care in their homes, with monitoring, diagnostic testing, and face-to-face contact with physicians and other health care professionals; it should be noted that this model is distinctly different from home nursing care for the administration of IV medications. Our results are concordant with reviews of HaH in other patient populations. A systematic review of 10 RCTs (n = 1333) involving general medical and surgical patients examined the effectiveness of HaH and demonstrated a significant 6-month mortality reduction in the HaH group, but found no significant difference in readmissions [14]. Although HaH did not improve HrQOL, functional or cognitive ability, patient satisfaction was improved while also reducing direct health care costs. Another systematic review of 8 RCTs (n = 870) involving patients presenting to the ED with acute exacerbations of COPD found that HaH significantly reduced readmissions, with a non-significant decrease in mortality [33]. HaH also reduced costs, but the quality of evidence for this outcome was low. A systematic review of 26 RCTs (n = 3967) found early discharge from hospital followed by HaH for general medical conditions increased patient satisfaction and decreased the risk of residential care at follow-up [13]. There was no difference in mortality and no clear reduction in cost.

Our search strategy was rigorous and thorough, was not restricted by language, and included abstracts and conference proceedings. However, we found only a limited number of studies that evaluated HaH in patients with HF. We were therefore limited by the available RCTs and observational studies when performing analyses. This restricted us from conducting meta-regression, assessing publication bias, or achieving the statistical power needed to draw reliable conclusions. Studies were also limited in methodological quality. Details regarding the intervention and RH were not always clearly provided, a known limitation of complex interventions [34]. Given the nature of the intervention, none of the studies blinded participants or clinicians. While this was less likely to have influenced objective outcomes (e.g. mortality), it could have influenced outcomes such as HrQOL. The observational studies did not account for background trends or confounders in the analysis, and varied in the intervention tested. We did not pool their results for meta-analysis. Furthermore, all studies were European, with 4 of 6 trials conducted in Spain, which could limit the generalizability our findings to other continents and health care systems.

We found that the HaH interventions varied across studies in the level of care provided, intensity of contact, and organization of healthcare professionals. Given the small number and variation between studies in the intervention tested, we were unable to determine which features of HaH may have been more effective than others. Outcome measures also varied, limiting the ability to pool results for meta-analysis. We tried to overcome this limitation by using the standardized mean difference (SMD) to pool studies that used different HrQOL scales [18]. However, while the SMD standardizes the units of measurement, it is influenced by sample size and heterogeneity of study populations [18,35]. Studies containing a more homogenous population and a larger sample size are more likely to have a smaller SD, which has the potential to artificially increase the SMD [35]. This can also falsely homogenize studies, limiting the detection of true heterogeneity with tests such as the I^2^ index [35]. Furthermore, the SMD is reported as SD units, and this is difficult to interpret and apply to individual patients [18,35].

### Future directions

Larger RCTs are required to determine the effect of HaH on clinical outcomes in HF. A consistent definition for HaH should be adopted [36]. The effect of HaH on health care utilization as well as patient, caregiver, and provider satisfaction also needs to be assessed, since these factors will determine the feasibility and sustainability of this complex model of care. Cost outcomes should measure direct and indirect total costs not only for the index hospitalization but also for follow-up care. Caregiver burden and HrQOL, as well as loss of income, should also be considered as outcomes.

HaH may be especially useful in patients with advanced HF who experience a relapsing and remitting course, with progressive decline in health and recurrent need for hospital-level care. In these patients, HaH may provide a unique opportunity to offer comprehensive care in the convenience of the patient’s home, improve patient and provider satisfaction, and conserve health care resources.

## Conclusions

The literature to date on HaH in HF patients is limited and based on small studies of modest quality that lack standardized definitions and interventions. In the context of these limitations, HaH appears to increase time to readmission, improve HrQOL, and reduce costs of index hospitalization compared to routine hospitalization in select patients with decompensated HF. HaH does not significantly reduce readmissions or mortality, but the present study is underpowered to detect a statistically significant difference in these outcomes. Larger clinical trials are warranted to assess the effect of HaH schemes on clinical outcomes and long-term costs to health care systems.

## Supporting Information

S1 PRISMA Checklist (DOC)

S1 Fig Time to initial readmission in days for HaH compared with inpatients in randomized controlled trials. (DOCX)

S2 Fig Length of index stay in days for HaH compared with inpatients in randomized controlled trials. (DOCX)

S1 Table Number of readmissions for substitutive care compared to RH. (DOCX)

S2 Table Comparison between substitutive care and RH for additional outcomes reported by single studies. (DOCX)

S3 Table MEDLINE search strategy. (DOCX)

S4 Table EMBASE search strategy. (DOCX)

S5 Table CINAHL search strategy. (DOCX)

S6 Table CENTRAL search strategy. (DOCX)

S7 Table Characteristics and risk of bias for included studies. (DOCX)
