# Supplementary figures and images for: Efficacy of Hospital at Home in Patients with Heart Failure: A Systematic Review and Meta-Analysis
Source: PLoS One. 2015 Jun 8;10(6):e0129282. doi: 10.1371/journal.pone.0129282 (PMC4460137; doi:10.1371/journal.pone.0129282)

|  |
| --- |


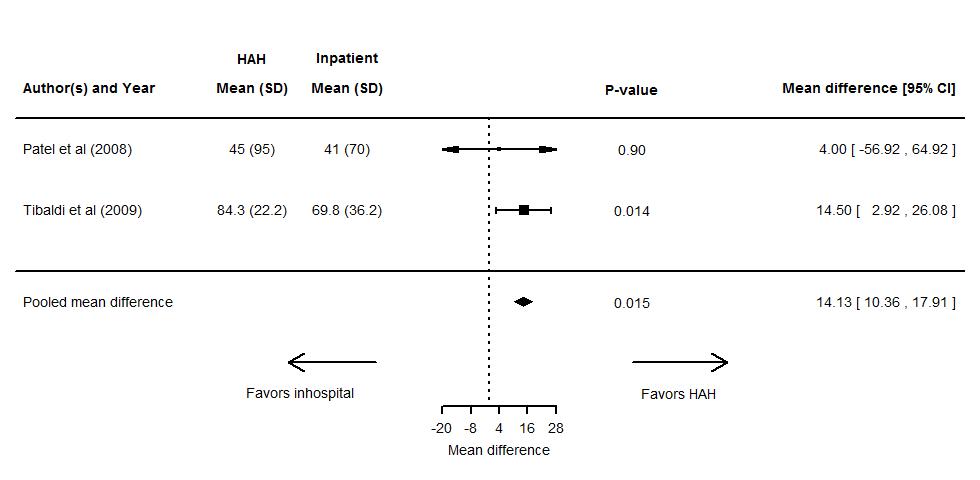


|  |
| --- |

I2 <1%
CI = confidence interval; HaH = hospital at home; SD = standard deviation

Supplement: S1 Fig — (DOCX) [file pone.0129282.s002.docx]

|  |
| --- |


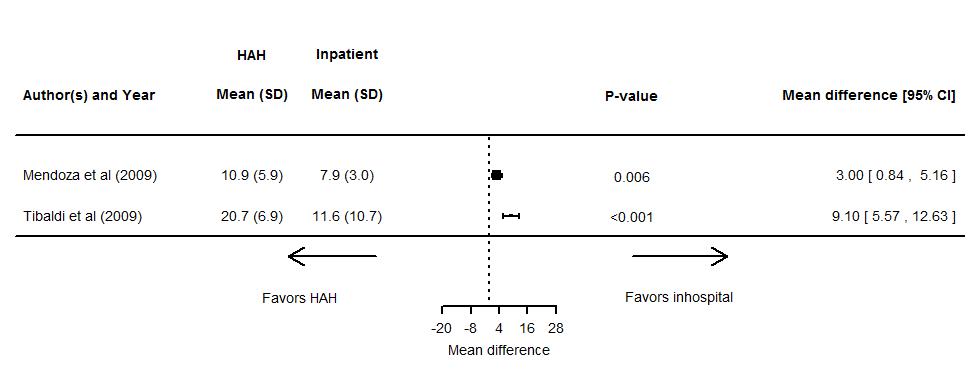


| I2 = 88% CI = confidence interval; HaH = hospital at home; SD = standard deviation |
| --- |

Supplement: S2 Fig — (DOCX) [file pone.0129282.s003.docx]
