# Supplementary material for: Efficacy of Hospital at Home in Patients with Heart Failure: A Systematic Review and Meta-Analysis
Source: PLoS One. 2015 Jun 8;10(6):e0129282. doi: 10.1371/journal.pone.0129282 (PMC4460137; doi:10.1371/journal.pone.0129282)
Supplement: S1 Table — (DOCX) [file pone.0129282.s004.docx]

**Table S1.** Number of readmissions for substitutive care compared to RH.

|  | | | | | |
| --- | --- | --- | --- | --- | --- |
| **Reference**  **(Study design)** | **Sample Size** | **Outcome** | **Substitutive care** | **Usual care** | **P value** |
| Mendoza 2009  (RCT) | 37 in HaH; 34 in RH | 12-month HF readmissions | 32* | 31* | NS |
| Roig 2006 (Observational) | 61 patients acted as their own control | All-cause readmissions in a mean (SD) follow-up of 11(10) months | 108 | 308 | NR |

*Not all readmissions were to hospital. In the HaH group, 25 readmissions were to hospital and 7 readmissions to HaH. In the control group, 29 readmissions were to hospital and 2 to HaH

HaH = hospital at home; HF = heart failure; NR = not reported; NS = not significant; RCT = randomized controlled trial; SD = standard deviation
