# Supplementary material for: Efficacy of Hospital at Home in Patients with Heart Failure: A Systematic Review and Meta-Analysis
Source: PLoS One. 2015 Jun 8;10(6):e0129282. doi: 10.1371/journal.pone.0129282 (PMC4460137; doi:10.1371/journal.pone.0129282)
Supplement: S2 Table — (DOCX) [file pone.0129282.s005.docx]

|  | | | | | |
| --- | --- | --- | --- | --- | --- |
| **Reference**  **(Study design)** | **Sample Size** | **Results** | | | |
|  |  | **Outcome** | **Substitutive care** | **RH** | **P value** |
| Mendoza 2009 (RCT) | 37 in HaH; 34 in RH | Combined 12-month mortality or readmission due to cardiovascular event; number (%) of patients | 20 (54%) | 19 (56%) | 0.88 |
| Roig 2006 (Observational) | 61 acted as their own control | Number (%) of patients with >3 readmissions per year | 7 (11%) | 42 (69%) | <0.001 |
|  |  | Mean cost of healthcare per patient-year (Euros) | 17585 | 19175 | NR |
| Tibaldi 2009 (RCT) | 48 in HaH; 53 in RH | Number (%) of institutionalizations into long-term care facility | 0 | 8 (16%) | NR |
|  |  | Mean daily cost of institutionalization into long-term care facility (Euros) | 0 | 157.12 | NR |
|  |  | Mean number of days spent in long-term facility | 0 | 26 | NR |
|  |  | 6-month mean (SD) change from baseline depression (Geriatric Depression Scale) | 1.48 (1.86) | 0.12 (3.36) | 0.020 |
|  |  | 6-month mean (SD) change from baseline cognitive status (Mini-Mental State Examination) | 0.07 (1.38) | 0.08 (1.36) | 0.97 |
|  |  | 6-month mean (SD) change from baseline nutritional status (Mini Nutritional Assessment) | -0.86 (1.12) | -0.27 (1.78) | 0.050 |
|  |  | Mean cost per patient per day (Euros) | 110.98 | 280.62 | NR |

**Table S2.** Comparison between substitutive care and RH for additional outcomes reported by single studies.

We made comparisons between groups using 6-month change from baseline and 12-month change from baseline

HaH = hospital at home; NR = not reported; NS = not significant; RH = routine hospitalization; SD = standard deviation
