# Supplementary material for: Efficacy of Hospital at Home in Patients with Heart Failure: A Systematic Review and Meta-Analysis
Source: PLoS One. 2015 Jun 8;10(6):e0129282. doi: 10.1371/journal.pone.0129282 (PMC4460137; doi:10.1371/journal.pone.0129282)
Supplement: S3 Table — (DOCX) [file pone.0129282.s006.docx]

Table S3. MEDLINE search strategy.

Database: Ovid MEDLINE (R) In-Process & Other Non-Indexed Citations and Ovid MEDLINE (R) 1946 to Present

Date: 05 June 2013  
Number of hits: 682

| **#** | **Searches** | **Results** |
| --- | --- | --- |
| 1 | exp Heart Failure/ | 81800 |
| 2 | (heart adj2 fail*).mp. [mp=title, abstract, original title, name of substance word, subject heading word, keyword heading word, protocol supplementary concept, rare disease supplementary concept, unique identifier] | 129418 |
| 3 | (cardiac adj fail*).mp. [mp=title, abstract, original title, name of substance word, subject heading word, keyword heading word, protocol supplementary concept, rare disease supplementary concept, unique identifier] | 9744 |
| 4 | or/1-3 | 135738 |
| 5 | Home Care Services, Hospital-Based/ or Home Care Agencies/ or Home Infusion Therapy/ or Home Nursing/ or Home Care Services/ | 36596 |
| 6 | home.mp. | 158722 |
| 7 | Mobile Health Units/ | 2857 |
| 8 | mobile health unit*.mp. | 2888 |
| 9 | House Calls/ | 2262 |
| 10 | house call*.mp. [mp=title, abstract, original title, name of substance word, subject heading word, keyword heading word, protocol supplementary concept, rare disease supplementary concept, unique identifier] | 2451 |
| 11 | or/5-10 | 162499 |
| 12 | exp hospitalization/ | 149068 |
| 13 | hospitaliz*.mp. [mp=title, abstract, original title, name of substance word, subject heading word, keyword heading word, protocol supplementary concept, rare disease supplementary concept, unique identifier] | 172238 |
| 14 | hospital-at-home.mp. | 274 |
| 15 | inpatients/ | 11804 |
| 16 | inpatient*.mp. | 65154 |
| 17 | exp Day Care/ | 4541 |
| 18 | (day care or after care).mp. [mp=title, abstract, original title, name of substance word, subject heading word, keyword heading word, protocol supplementary concept, rare disease supplementary concept, unique identifier] | 12874 |
| 19 | ((hospital or patient) adj2 (admission or readmission)).mp. | 41913 |
| 20 | ((early or hospital or patient) adj1 discharge).mp. | 31609 |
| 21 | or/12-20 | 314810 |
| 22 | 4 and 11 and 21 | 704 |
| 23 | limit 22 to yr="1990-Current" | 682 |
