# Supplementary material for: Efficacy of Hospital at Home in Patients with Heart Failure: A Systematic Review and Meta-Analysis
Source: PLoS One. 2015 Jun 8;10(6):e0129282. doi: 10.1371/journal.pone.0129282 (PMC4460137; doi:10.1371/journal.pone.0129282)
Supplement: S4 Table — (DOCX) [file pone.0129282.s007.docx]

Table S4. EMBASE search strategy.

**Database: EMBASE** 1980 to 2013 Week 21
**Date:** 05 June 2013
**Number of hits**: 2076

| **#** | **Searches** | **Results** |
| --- | --- | --- |
| 1 | exp heart failure/ | 263695 |
| 2 | exp congestive heart failure/ | 73268 |
| 3 | ((cardiac adj fail*) or (heart adj2 fail*)).mp. [mp=title, abstract, subject headings, heading word, drug trade name, original title, device manufacturer, drug manufacturer, device trade name, keyword] | 221775 |
| 4 | or/1-3 | 297616 |
| 5 | exp home care/ | 50565 |
| 6 | Home Care Services, Hospital-Based/ or Home Care Agencies/ or Home Infusion Therapy/ or Home Nursing/ or Home Care Services/ | 80505 |
| 7 | home.mp. | 218648 |
| 8 | mobile health units/ | 20356 |
| 9 | mobile health unit*.mp. | 56 |
| 10 | professional practice/ | 47824 |
| 11 | house call*.mp. [mp=title, abstract, subject headings, heading word, drug trade name, original title, device manufacturer, drug manufacturer, device trade name, keyword] | 598 |
| 12 | or/5-11 | 317853 |
| 13 | hospital admission/ or hospital care/ or hospital discharge/ or hospital patient/ or hospital readmission/ or hospital services/ or hospitalization/ or length of stay/ | 424635 |
| 14 | hospitaliz*.mp. [mp=title, abstract, subject headings, heading word, drug trade name, original title, device manufacturer, drug manufacturer, device trade name, keyword] | 295471 |
| 15 | hospital-at-home.mp. | 358 |
| 16 | inpatients/ | 62206 |
| 17 | inpatient*.mp. [mp=title, abstract, subject headings, heading word, drug trade name, original title, device manufacturer, drug manufacturer, device trade name, keyword] | 80535 |
| 18 | exp day care/ | 9095 |
| 19 | (day care or after care).mp. [mp=title, abstract, subject headings, heading word, drug trade name, original title, device manufacturer, drug manufacturer, device trade name, keyword] | 13466 |
| 20 | ((hospital or patient) adj2 (admission or readmission)).mp. | 124289 |
| 21 | ((early or hospital or patient) adj1 discharge).mp. | 67004 |
| 22 | or/13-21 | 566616 |
| 23 | 4 and 12 and 22 | 2107 |
| 24 | limit 23 to yr="1990-Current" | 2076 |
