# Supplementary material for: Efficacy of Hospital at Home in Patients with Heart Failure: A Systematic Review and Meta-Analysis
Source: PLoS One. 2015 Jun 8;10(6):e0129282. doi: 10.1371/journal.pone.0129282 (PMC4460137; doi:10.1371/journal.pone.0129282)
Supplement: S5 Table — (DOCX) [file pone.0129282.s008.docx]

Table S5. CINAHL search strategy.

**Database: CINAHL**
**Date:** 05 June 2013
**Number of hits**: 431

| **#** | **Query** | **Limiters/Expanders** | **Last Run Via** | **Results** | **Action** |
| --- | --- | --- | --- | --- | --- |
| S30 | S4 AND S11 AND S28 | Limiters - Published Date from: 19900101-20130631 Search modes - Boolean/Phrase | Interface - EBSCOhost Search Screen - Advanced Search Database - CINAHL | 431 |  |
| S29 | S4 AND S11 AND S28 | Search modes - Boolean/Phrase | Interface - EBSCOhost Search Screen - Advanced Search Database - CINAHL | 435 |  |
| S28 | S12 OR S13 OR S14 OR S15 OR S16 OR S17 OR S18 OR S19 OR S20 OR S21 OR S22 OR S23 OR S24 OR S25 OR S26 OR S27 | Search modes - Boolean/Phrase | Interface - EBSCOhost Search Screen - Advanced Search Database - CINAHL | 119,455 |  |
| S27 | patient N1 discharge | Search modes - Boolean/Phrase | Interface - EBSCOhost Search Screen - Advanced Search Database - CINAHL | 9,227 |  |
| S26 | hospital N1 discharge | Search modes - Boolean/Phrase | Interface - EBSCOhost Search Screen - Advanced Search Database - CINAHL | 4,641 |  |
| S25 | early N1 discharge | Search modes - Boolean/Phrase | Interface - EBSCOhost Search Screen - Advanced Search Database - CINAHL | 1,211 |  |
| S24 | patient N2 readmission | Search modes - Boolean/Phrase | Interface - EBSCOhost Search Screen - Advanced Search Database - CINAHL | 59 |  |
| S23 | patient N2 admission | Search modes - Boolean/Phrase | Interface - EBSCOhost Search Screen - Advanced Search Database - CINAHL | 7,238 |  |
| S22 | hospital N2 readmission | Search modes - Boolean/Phrase | Interface - EBSCOhost Search Screen - Advanced Search Database - CINAHL | 602 |  |
| S21 | hospital N2 admission | Search modes - Boolean/Phrase | Interface - EBSCOhost Search Screen - Advanced Search Database - CINAHL | 3,905 |  |
| S20 | after care | Search modes - Boolean/Phrase | Interface - EBSCOhost Search Screen - Advanced Search Database - CINAHL | 5,329 |  |
| S19 | day care | Search modes - Boolean/Phrase | Interface - EBSCOhost Search Screen - Advanced Search Database - CINAHL | 4,003 |  |
| S18 | (MH "After Care") | Search modes - Boolean/Phrase | Interface - EBSCOhost Search Screen - Advanced Search Database - CINAHL | 5,219 |  |
| S17 | (MH "Day Care") | Search modes - Boolean/Phrase | Interface - EBSCOhost Search Screen - Advanced Search Database - CINAHL | 1,697 |  |
| S16 | inpatient* | Search modes - Boolean/Phrase | Interface - EBSCOhost Search Screen - Advanced Search Database - CINAHL | 63,072 |  |
| S15 | (MH "Inpatients") | Search modes - Boolean/Phrase | Interface - EBSCOhost Search Screen - Advanced Search Database - CINAHL | 52,244 |  |
| S14 | hospital-at-home | Search modes - Boolean/Phrase | Interface - EBSCOhost Search Screen - Advanced Search Database - CINAHL | 916 |  |
| S13 | hospitaliz* | Search modes - Boolean/Phrase | Interface - EBSCOhost Search Screen - Advanced Search Database - CINAHL | 33,329 |  |
| S12 | (MH "Hospitalization") OR (MH "Length of Stay") OR (MH "Patient Admission") OR (MH "Patient Discharge") OR (MH "Early Patient Discharge") OR (MH "Readmission") | Search modes - Boolean/Phrase | Interface - EBSCOhost Search Screen - Advanced Search Database - CINAHL | 38,058 |  |
| S11 | S5 OR S6 OR S7 OR S8 OR S9 OR S10 | Search modes - Boolean/Phrase | Interface - EBSCOhost Search Screen - Advanced Search Database - CINAHL | 88,629 |  |
| S10 | house call* | Search modes - Boolean/Phrase | Interface - EBSCOhost Search Screen - Advanced Search Database - CINAHL | 405 |  |
| S9 | (MH "Home Visits") | Search modes - Boolean/Phrase | Interface - EBSCOhost Search Screen - Advanced Search Database - CINAHL | 3,235 |  |
| S8 | mobile health unit* | Search modes - Boolean/Phrase | Interface - EBSCOhost Search Screen - Advanced Search Database - CINAHL | 994 |  |
| S7 | (MH "Mobile Health Units") | Search modes - Boolean/Phrase | Interface - EBSCOhost Search Screen - Advanced Search Database - CINAHL | 991 |  |
| S6 | "home" | Search modes - Boolean/Phrase | Interface - EBSCOhost Search Screen - Advanced Search Database - CINAHL | 87,458 |  |
| S5 | (MH "Home Health Agencies") OR (MH "Home Health Care") OR (MH "Home Intravenous Therapy") OR (MH "Home Nursing, Professional") OR (MH "Home Rehabilitation") | Search modes - Boolean/Phrase | Interface - EBSCOhost Search Screen - Advanced Search Database - CINAHL | 23,511 |  |
| S4 | S1 OR S2 OR S3 | Search modes - Boolean/Phrase | Interface - EBSCOhost Search Screen - Advanced Search Database - CINAHL | 21,356 |  |
| S3 | cardiac N1 fail* | Search modes - Boolean/Phrase | Interface - EBSCOhost Search Screen - Advanced Search Database - CINAHL | 600 |  |
| S2 | heart N2 fail* | Search modes - Boolean/Phrase | Interface - EBSCOhost Search Screen - Advanced Search Database - CINAHL | 21,071 |  |
| S1 | (MH "Heart Failure+") | Search modes - Boolean/Phrase | Interface - EBSCOhost Search Screen - Advanced Search Database - CINAHL | 16,321 |  |
