# Supplementary material for: Efficacy of Hospital at Home in Patients with Heart Failure: A Systematic Review and Meta-Analysis
Source: PLoS One. 2015 Jun 8;10(6):e0129282. doi: 10.1371/journal.pone.0129282 (PMC4460137; doi:10.1371/journal.pone.0129282)

Table S6. CENTRAL search strategy.

**Database: Cochrane Central Register of Controlled Trials** May 2013

**Date:** 05 June 2013

**Number of hits**: 200


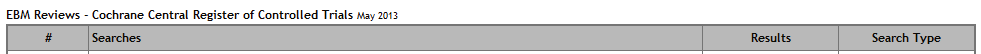

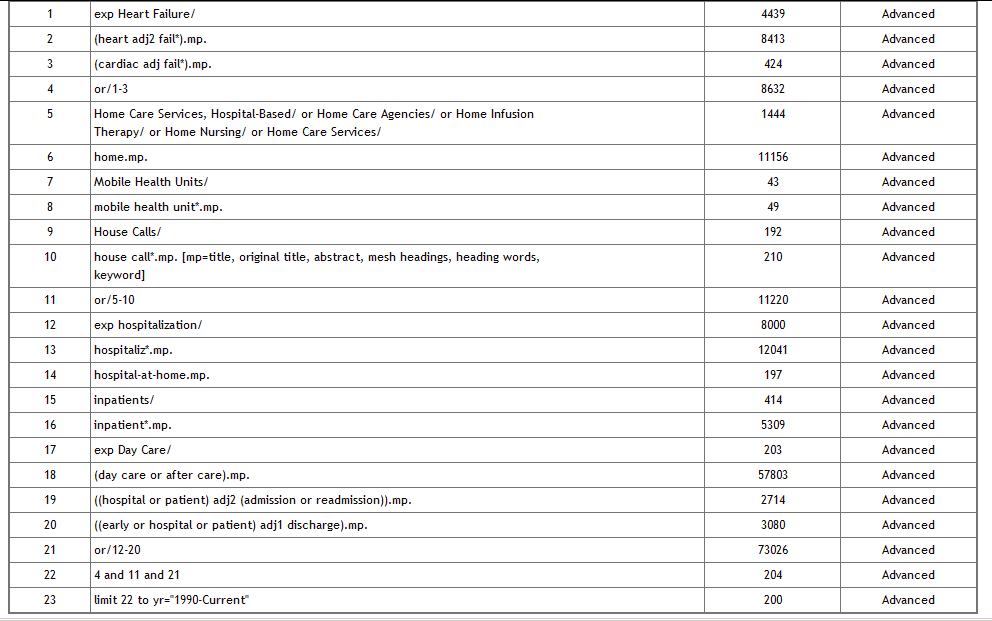

Supplement: S6 Table — (DOCX) [file pone.0129282.s009.docx]
