# Supplementary material for: Efficacy of Hospital at Home in Patients with Heart Failure: A Systematic Review and Meta-Analysis
Source: PLoS One. 2015 Jun 8;10(6):e0129282. doi: 10.1371/journal.pone.0129282 (PMC4460137; doi:10.1371/journal.pone.0129282)
Supplement: S7 Table — (DOCX) [file pone.0129282.s010.docx]

**Table S7.** Characteristics and risk of bias for included studies with references.

**Bechich 2000**^1^

| Methods | Unicenter before-after study **Recruitment:** Study staff recruited patients diagnosed with decompensated HF presenting to ED (Nov 1996-Oct 1998) **Follow up duration/frequency:** 90 days before and after intervention | |
| --- | --- | --- |
| Participants | **Location:** Hospital del Sagrat Cor de Barcelona, Spain  **Participants:** 110 patients  **Demographics:** Mean age (SD) 84.7 (6.1), male sex NR, marital status and education NR  **Baseline NYHA functional class:** NR  **Baseline systolic function:** NR  **Inclusion criteria:** Age ˃70 diagnosed with HF using Framingham criteria and radiologic evidence. Patients must have lived within the hospital`s catchment, had a telephone, hygienic conditions, and support at home  **Exclusion criteria:** Malignant arrhythmia, resistance to initial treatment in ED, acute coronary syndrome in previous 3 months | |
| Intervention(s) | Patients were categorized into 5 levels of care:  5) Nursing visits 3X/week and medical visits 2X/week  4) Nursing visit 3X/week and medical visit 1X/week  3) Nursing visit 1X/week and telephone contact 1X/week 2) Call scheduled weekly  1) Unscheduled telephone contact.  Provided elements included: education about self-management and detecting signals of decompensation, compliance with diet, and compliance with medication. Patients had telephone access to hospital-at-home staff between 8 a.m. and 8 p.m., and could call the hospital emergency services outside of those hours | |
| Outcome(s) | - Readmission - ED visits - Functional status (Barthel index) - Satisfaction with care (only for intervention) | |
| Note(s) |  | |
| ***Risk of bias*** | | |
| **Bias** | **Authors’ judgement** | **Support for judgement** |
| Random sequence generation (selection bias) | Inadequate | Non-randomized design |
| Allocation concealment (selection bias) | Inadequate | Study personnel knew which patients would be allocated to hospital-at-home as this was the only group assignment |
| Blinding of participants and assessors (performance and detection bias): objective outcomes | Adequate | No blinding, but the outcome and outcome assessment are unlikely to be influenced by the lack of blinding |
| Blinding of participants and assessors (performance and detection bias): subjective outcomes | Inadequate | No blinding, and the outcome or outcome assessment is likely influenced by the lack of blinding |
| Incomplete outcome data (attrition bias): all outcomes | Adequate | No patients dropped out of the study |
| Selective reporting (reporting bias) | Adequate | Not every category of the satisfaction survey is reported, but the review authors judged that this was not sufficient enough to have an impact on the results |
| Other bias | Unclear | Confounders were not assessed |

**de Zuazu 2003**^2^

| Methods | Unicenter before-after study **Recruitment:** Diagnosis of HF from the ED, ward, or outpatient clinic (Jan 1999-Dec 2001) **Follow up duration/frequency:** 90 days before and after intervention | |
| --- | --- | --- |
| Participants | **Location:** Txagorritxu University Hospital, Vitoria-Gasteiz, Spain  **Participants:** 158 patients in intervention group acted as their own controls for the main outcomes; 1358 patients receiving inpatient hospital care acted as concurrent controls for other factors (see Notes)  **Demographics:**  Intervention group: Mean age (SD) 78.1 (6.77), male sex 42%, marital status and education NR  Concurrent control group: Mean age (SD) 75.8 (10.5), male sex 49%, marital status and education NR  **Baseline NYHA functional class:** I-II 26, III 103, IV 29  **Baseline systolic function:** NR  **Inclusion criteria:** Known diagnosis of HF for at least 2 years with NYHA III-IV functional class symptoms. If recruited from ward or clinic, patients may have end-stage HF  **Exclusion criteria:** Sudden deterioration of heart function, hemodynamics, or biochemistry, outside hours of operation for intervention, alcoholism, active psychiatric issues | |
| Intervention(s) | Nurses visit daily, doctors visit at least every other day.  Blood work, electrocardiogaphy, radiography performed at home. Intervention ran between 0800h – 2100h. Patients were to contact emergency services off-hours | |
| Outcome(s) | - ED visits - Readmissions - Average length of stay in care - All-cause mortality | |
| Note(s) | In this before-after study, patients served as their own controls for the outcomes of interest (ED visits and readmission). Measurements were taken 90 days before the intervention (hospital-at-home), control (usual hospital care), and 90 days after hospital-at-home. For other baseline factors (deaths, length of stay, age, sex), comparisons were made between the 158 hospital-at-home patients to 1358 concurrent controls receiving usual hospital care | |
| ***Risk of bias*** | | |
| **Bias** | **Authors’ judgement** | **Support for judgement** |
| Random sequence generation (selection bias) | Inadequate | Non-randomized design |
| Allocation concealment (selection bias) | Inadequate | Study personnel knew which patients would be allocated to hospital-at-home as this was the only group assignment |
| Blinding of participants and assessors (performance and detection bias): objective outcomes | Adequate | No blinding, but the outcome and outcome assessment are unlikely to be influenced by the lack of blinding |
| Blinding of participants and assessors (performance and detection bias): subjective outcomes | Inadequate | No blinding, and the outcome or outcome assessment is likely influenced by the lack of blinding |
| Incomplete outcome data (attrition bias): all outcomes | Adequate | No patients dropped out of the study |
| Selective reporting (reporting bias) | Adequate | All expected outcomes are reported |
| Other bias | Unclear | 29 patients were sent back to hospital care after receiving hospital-at-home, which may have contaminated the results. Also, confounders were not assessed |

**Mendoza 2009**^3^

| Methods | Multicenter RCT **Recruitment:** A monitoring committee screened patients who presented in the ED with decompensated HF (May 2006-Mar 2007)  **Follow up duration/frequency:** Patients contacted at 1,3,6, and 12 months | |
| --- | --- | --- |
| Participants | **Location:** Txagorritxu University Hospital, Vitoria-Gasteiz, Spain  **Participants:**  Intervention group 37  Control group 34  **Demographics:**  Intervention group: Mean age (SD) 78.1 (6.2), male sex 49%, marital status and education NR  Control group: Mean age (SD) 79.9 (6.3), male sex 71%, marital status and education NR  **Baseline NYHA functional class:**  Intervention group: II 19, III 18  Control group: II 23, III 11  **Baseline systolic function:**  Intervention group: LVEF ≥ 45% 23, LVEF < 45% 14  Control group: LVEF ≥ 45% 24, LVEF < 45% 10  **Inclusion criteria:** Age ˃65 who had diagnosis of HF at least 12 months prior to presentation with baseline NYHA II-III functional class  **Exclusion criteria:** Resistance to initial treatment in ED, admission to hospital 2 months prior to presentation for decompensated HF or acute coronary syndrome, active psychiatric issues, active tuberculosis, poor prognosis with life expectancy ˂6 months, no supervision at home, no telephone access, beyond hospital catchment (˃10 km) | |
| Intervention(s) | Specialist nurse visits daily, physician visits daily or every other day  Blood work and electrocardiograms performed in the home with equal access to radiography and echocardiography done in hospital. Patients were then referred to their primary care provider if recovered, or transferred to the hospital if deteriorated at home | |
| Outcome(s) | - Number of patients with HF readmission within 12 months - Combined 12-month mortality or readmission due to HF or other cardiovascular cause - 12-month all-cause death - Average length of stay in care - Functional status (Barthel index) - Health-related quality of life (SF-36, physical and mental) - Costs | |
| Note(s) | Funding: Caja Vital Kutxa (no conflict of interest declared)  Another article (Garcia-Soletos 2013) from the same study, but with a longer follow-up period, was published in 2013. This article reported additional data on: functional status (Barthel index), and health-related quality of life (SF-36, EQ-5D, and the Minnesota Living with Heart Failure Questionnaire) | |
| ***Risk of bias*** | | |
| **Bias** | **Authors’ judgement** | **Support for judgement** |
| Random sequence generation (selection bias) | Low risk | Quote: “Once the patient had signed the informed consent form, they were randomly assigned (1:1) to one of the intervention groups according to an externally generated sequence, which was hidden from the clinicians until the patient had given consent to participate.” |
| Allocation concealment (selection bias) | Unclear | Although the authors of this study state that allocation concealment is hidden from study personnel, there is not sufficient detail describing the method of allocation concealment |
| Blinding of participants and assessors (performance and detection bias): objective outcomes | Low risk | No blinding, but the outcome and outcome assessment are unlikely to be influenced by the lack of blinding |
| Blinding of participants and assessors (performance and detection bias): subjective outcomes | High risk | No blinding, and the outcome or outcome assessment is likely influenced by the lack of blinding |
| Incomplete outcome data (attrition bias): all outcomes | High risk | Two dropouts (5.4%) in the treatment group, and 7 (20.6%) dropouts in the control group. Of note, the reasons for dropout were not described for each group individually |
| Selective reporting (reporting bias) | Low risk | All expected outcomes reported |
| Other bias | Unclear risk | Lack of standard protocol may lead to contamination. Two patients initially randomized to usual inpatient care were re-admitted to the hospital-at-home, not the hospital. Furthermore, seven patients initially randomized to hospital-at-home were re-admitted to hospital-at-home, not the hospital. All other readmissions were done in the hospital |

**Patel 2008**^4^

| Methods | Unicenter RCT **Recruitment:** Identification and screening of patients diagnosed with decompensated HF within 24 hours (amended after 1 year to 48 hours) of presentation to ED, ward, or outpatient clinic (Apr 2004-May 2006) **Follow up duration/frequency:** Patients contacted at 1,4,8, and 12 months | |
| --- | --- | --- |
| Participants | **Location:** Sahlgrenska University Hospital, Östra Göteborg, Sweden  **Participants:**  Intervention group 13  Control group 18  **Demographics:**  Intervention group: Mean age (SD) 77 (10), male sex 46%, divorced 2, single 1, widowed 7, education ≥ 9 years 1  Control group: Mean age (SD) 78 (8), male sex 83%, divorced 3, single 2, widowed 5, education ≥ 9 years 8  **Baseline NYHA functional class:**  Intervention group: III 13  Control group: II 1, III 16, IV 1  **Baseline systolic function:**  Intervention group: Mean LVEF (SD) 36% (13)  Control group: Mean LVEF (SD) 33% (12)  **Inclusion criteria:** Previous diagnosis of HF by European Society of Cardiology Guidelines with clinical signs and symptoms of decompensated HF and NYHA functional class II – IV symptoms on presentation  **Exclusion criteria:** New HF diagnosis, worsening symptoms ˂3 days, malignant arrhythmia, biochemical or hemodynamic instability, other comorbidities requiring hospitalization, institutionalized, unable to follow instructions, other comorbidities requiring hospitalization | |
| Intervention(s) | Specialist nurse visits daily or every other day for 5-7 days  Education for self-management and involvement of allied health professionals as necessary. After consultation with a cardiologist, nurses administered intravenous diuretics following a combination clinical and biochemical (electrolytes, renal function drawn at home). Nurses and cardiologists were always available by telephone if necessary. Home visits terminated with symptom improvement, falling weight, resolution of pulmonary crackles and edema above the ankle. If necessary, patients were discharged to heart failure clinic for follow-up. | |
| Outcome(s) | - Time consumed for index episode - Physician time consumed - Number of home visits (intervention group) - Number of visits to heart failure clinic - Time to first readmission - Mean number of HF-related ED visits   - Proportion of these patients hospitalized     - Time in hospital for these patients - Probability of hospitalization (Kaplan-Meier analysis) - Weight - NYHA class - NT-proBNP - Proportion of patients using various drugs (ACE inhibitors, beta-blockers, spironolactone, diuretics) - Health related quality of life and quality-adjusted life-years (Kansas City Cardiomyopathy Questionnaire, SF-36, EuroQol five-dimension questionnaire of HRQL with the visual analogue scale, and the utility-based standard gamble) - Costs - Cost-utility | |
| Note(s) | Not all patients completed the study. Two patients died in each of the intervention and control groups. The review authors included these deaths in the analysis for all-cause mortality | |
| ***Risk of bias*** | | |
| **Bias** | **Authors’ judgement** | **Support for judgement** |
| Random sequence generation (selection bias) | Low risk | Quote: “Patients were randomised using a random number generator to either home care (HC) under the direction of a specialist nurse or to hospital admission/conventional care (CC).” |
| Allocation concealment (selection bias) | Unclear risk | Not enough information to ascertain that allocation to treatment or control group was hidden |
| Blinding of participants and assessors (performance and detection bias): objective outcomes | Low risk | No blinding, but the outcome and outcome assessment are unlikely to be influenced by the lack of blinding |
| Blinding of participants and assessors (performance and detection bias): subjective outcomes | High risk | No blinding, and the outcome or outcome assessment is likely influenced by the lack of blinding |
| Incomplete outcome data (attrition bias): all outcomes | High risk | Three (17%) of the control group patients withdrew consent. There were no patient dropouts in the treatment group. Sensitivity analysis was used with the last value carried forward for the patients that withdrew consent, but review authors still judged this as a high risk due to the difference in magnitude of withdrawal between the groups |
| Selective reporting (reporting bias) | Low risk | All expected outcomes are reported |
| Other bias | Low risk | There were significantly more males and more educated participants in the control group, but review authors judged this to be insufficient to have an impact on the results, especially given the small sample size |

**Roig 2006**^5^

| Methods | Unicenter before-after study **Recruitment:** Patients with end-stage HF recruited from heart failure clinic to a day hospital (Jan 1998-Dec 2004) **Follow up duration/frequency:** Mean (SD) 11 (10) months | |
| --- | --- | --- |
| Participants | **Location:** Spain  **Participants:** 61 patients  **Demographics:** Mean age (SD) 64 (13), male sex 92%, marital status and education NR  **Baseline NYHA functional class:** III 23, IV 38  **Baseline systolic function:** Mean LVEF (SD) 23% (6)  **Inclusion criteria:** End-stage HF requiring palliative intravenous inotropic support  **Exclusion criteria:** NR | |
| Intervention(s) | In the first year, patients attended a day hospital with supervision by a specialized nurse and cardiologist. Depending on stability, patients were asked to return once a month to twice weekly. Blood work and administration of intravenous diuretics or inotropes (via Portacath for palliative patients) occurred.  In the second year, specialized nurses visited patients' homes (18 of 61 patients) to administer intravenous diuretics or inotropes after telephone consultation with a cardiologist | |
| Outcome(s) | - All-cause mortality - Total all-cause readmissions - Mean all-cause readmissions - Time in hospital - Number of visits to ED - Number of patients ˃3 readmissions per year - Mean cost of healthcare per patient-year | |
| Note(s) | This study compared outcomes after implementation of the specialized care program to outcomes a year before inclusion in the program | |
| ***Risk of bias*** | | |
| **Bias** | **Authors’ judgement** | **Support for judgement** |
| Random sequence generation (selection bias) | Inadequate | Non-randomized design |
| Allocation concealment (selection bias) | Inadequate | Study personnel knew which allocation as there was only one group |
| Blinding of participants and assessors (performance and detection bias): objective outcomes | Adequate | No blinding, but the outcome and outcome assessment are unlikely to be influenced by the lack of blinding |
| Blinding of participants and assessors (performance and detection bias): subjective outcomes | Inadequate | No blinding, and the outcome or outcome assessment is likely influenced by the lack of blinding |
| Incomplete outcome data (attrition bias): all outcomes | Adequate | Only 2 patients (3%) were lost to follow-up. Review authors judged this as a low risk |
| Selective reporting (reporting bias) | Adequate | All expected outcomes are reported |
| Other bias | Unclear | Unclear whether the study protocol was standardized at the two time points of outcome assessment: after implementation of the specialized care program, and a year prior to being included in the program Also, confounders were not assessed |

**Tibaldi 2009**^6^

| Methods | Unicenter RCT **Recruitment:** Patients enrolled within 12 to 24 hours after presenting to ED with decompensated HF (Apr 1 2004-Apr 31 2005) **Follow up duration/frequency:** 6 months | |
| --- | --- | --- |
| Participants | **Location:** San Giovanni Battista Hospital of Torino, Italy  **Participants:**  Intervention group 48  Control group 53  **Demographics:**  Intervention group: Mean age (SD) 82.2 (5.2), male sex 46%, married 22, family support at home 48, schooling <5 years 33  Control group: Mean age (SD) 80.1 (4.9), male sex 57%, married 24, family support at home 53, schooling <5 years 36  **Baseline NYHA functional class:** III 66, IV 35 (entire sample, with no significant difference between groups)  **Baseline systolic function:** LVEF <40% in 40% (entire sample, with no significant difference between groups)  **Inclusion criteria:** Age ˃75 with a previous diagnosis of Stage C HF by American Heart Association classification who presented to ED with decompensated HF and NYHA functional class III-IV symptoms  **Exclusion criteria:** New HF diagnosis, need for intensive care or surgery, severe dementia, renal impairment, hepatic failure, terminal malignant illness, lack of family and social support | |
| Intervention(s) | Nursing and physician visits at home daily initially to every 2 to 3 days or less. A team consisting of 4 geriatricians, 13 nurses, 3 physiotherapists, 1 social worker, and 1 counselor met daily to discuss patients' needs  Investigations and treatments included: pulse oximetry, spirometry, electrocardiography, echocardiography, Doppler ultrasonography, ambulatory electrocardiography, arterial blood pressure monitoring, oral and intravenous medication administration, oxygen therapy, blood transfusions, central venous access, surgical treatment of pressure sores. Protocols were in place to prevent nosocomial infections and bed sores.  Patients also received education for self-management, physical therapy, occupational therapy, and counseling. Urgent home visits were available if required. | |
| Outcome(s) | - 6-month all-cause mortality - Number of all-cause readmissions in 6 months - Time between discharge and first readmission - Length of stay in care during index episode - Length of stay in care of first readmission - Institutionalization to a long-term facility - Functional status - Depression - Cognitive status - Nutritional status - Costs | |
| Note(s) | Intention to treat principle used | |
| ***Risk of bias*** | | |
| **Bias** | **Authors’ judgement** | **Support for judgement** |
| Random sequence generation (selection bias) | Low risk | Quote: “…by the use of a set of computer-generated random numbers in a 1:1 ratio” |
| Allocation concealment (selection bias) | Low risk | Quote: “The allocation sequence was unknown to any of the investigators and was contained in a set of sealed envelopes, each bearing on the outside only the name of the hospital and a number, which was opened after the acceptance of the patient.” |
| Blinding of participants and assessors (performance and detection bias): objective outcomes | Low risk | No blinding, but the outcome and outcome assessment are unlikely to be influenced by the lack of blinding |
| Blinding of participants and assessors (performance and detection bias): subjective outcomes | High risk | No blinding, and the outcome or outcome assessment is likely influenced by the lack of blinding |
| Incomplete outcome data (attrition bias): all outcomes | Low risk | Two patients are lost to follow-up in both the treatment and control group |
| Selective reporting (reporting bias) | Low risk | All expected outcomes are reported |
| Other bias | Low risk | The treatment group had a slightly older population (p = 0.04), but review authors judged this to be insufficient to have a magnitude on the results |

**References**

1. Bechich S, Sort Granja D, Arroyo Mateo X, Delas Amat J, Rosell Abaurrea F (2000) Effect of home hospitalization in the reduction of traditional hospitalization and frequency of emergencies in heart failure. Rev Clin Esp 200: 310-314.

2. de Zuazu HMR, de los Cobos JR, Basurto EA, Ruiz JMC, Borau FA, et al. (2003) Treatment of congestive heart failure in the setting of hospital at home. Study of 158 patients. Med Clin 120: 405-407.

3. Mendoza H, Martin MJ, Garcia A, Aros F, Aizpuru F, et al. (2009) 'Hospital at home' care model as an effective alternative in the management of decompensated chronic heart failure. Eur J Heart Fail 11: 1208-1213.

4. Patel H, Shafazand M, Ekman I, Hojgard S, Swedberg K, et al. (2008) Home care as an option in worsening chronic heart failure -- a pilot study to evaluate feasibility, quality adjusted life years and cost-effectiveness. Eur J Heart Fail 10: 675-681.

5. Roig E, Perez-Villa F, Cuppoletti A, Castillo M, Hernandez N, et al. (2006) Specialized care program for end-stage heart failure patients. Initial experience in a heart failure unit. Rev Esp Cardiol 59: 109-116.

6. Tibaldi V, Isaia G, Scarafiotti C, Gariglio F, Zanocchi M, et al. (2009) Hospital at home for elderly patients with acute decompensation of chronic heart failure: a prospective randomized controlled trial. Arch Intern Med 169: 1569-1575.
